# Hepatoprotective and Antiviral Efficacy of *Acacia mellifera* Leaves Fractions against Hepatitis B Virus

**DOI:** 10.1155/2015/929131

**Published:** 2015-04-09

**Authors:** Ahmed H. Arbab, Mohammad K. Parvez, Mohammed S. Al-Dosari, Adnan J. Al-Rehaily, Mohammed Al-Sohaibani, Elwaleed E. Zaroug, Mansour S. AlSaid, Syed Rafatullah

**Affiliations:** ^1^Department of Pharmacognosy, College of Pharmacy, King Saud University, Riyadh 11451, Saudi Arabia; ^2^Department of Pharmacognosy, College of Pharmacy, Omdurman Islamic University, Khartoum 14415, Sudan; ^3^Department of Pathology, King Khalid University Hospital, King Saud University, Riyadh 11461, Saudi Arabia; ^4^Medicinal, Aromatic and Poisonous Plants Research Center, College of Pharmacy, King Saud University, Riyadh 11451, Saudi Arabia

## Abstract

The present study investigated the hepatoprotective and anti-HBV efficacy of *Acacia mellifera* (*AM*) leaves extracts. The crude ethanolic-extract, including organic and aqueous fractions, were tested for cytotoxicity on HepG2 and HepG2.2.15 cells (IC_50_ = 684 *μ*g/mL). Of these, the ethyl acetate and aqueous fractions showed the most promising, dose-dependent hepatoprotection in DCFH-toxicated cells at 48 h. In CCl_4_-injured rats, oral administration of *AM* ethanol extract (250 and 500 mg/kg·bw) for three weeks significantly normalized the sera aminotransferases, alkaline phosphatase, bilirubin, cholesterol, triglycerides, and lipoprotein levels and elevated tissue nonprotein sulphydryl and total protein. The histopathology of dissected livers also revealed that *AM* cured the tissue lesions. The phytochemical screening of the fractions showed presence of alkaloids, flavonoids, tannins, sterols, and saponins. Further, anti-HBV potential of the fractions was evaluated on HepG2.2.15 cells. Of these, the n-butanol and aqueous fractions exhibited the best inhibitory effects on HBsAg and HBeAg expressions in dose- and time-dependent manner. Taken together, while the ethyl acetate and aqueous fractions exhibited the most promising antioxidant/hepatoprotective and anti-HBV activity, respectively, the n-butanol partition showed both activities. Therefore, the therapeutic potential of *AM* extracts warrants further isolation of the active principle(s) and its phytochemical as well as biological studies.

## 1. Introduction

Liver injury accounts for approximately one-half of the cases of hepatic failures, including all forms of acute and chronic liver diseases [1]. In most of such cases, toxins and drugs are involved in oxidative stress-induced hepatotoxicity [2, 3]. Liver infection with hepatotropic viruses, including hepatitis viruses, is characterized by acute and chronic hepatitis, fibrosis, cirrhosis, and hepatocellular carcinomas. Of these, hepatitis B virus (HBV) infection continues to be an important cause of morbidity and mortality worldwide [4]. Although there are many effective therapeutic drugs available, they have certain limitations. While interferon (IFN-*α*) has a high incidence of adverse effects and nonresponse, long-term therapy with nucleos(t)ide analogues has risks of emergence of drug-resistant viral mutants [5]. Therefore, many effective natural or plant products have been investigated against hepatotoxin-induced liver damages [6–10]. In addition, numerous active phytoproducts or phytochemicals (flavonoids, polyphenolic tannins, terpenoids, lignans, saponins, alkaloids, and anthraquinones) of diverse geographic origin and based on local cultural practices have been also reported effective against HBV infections* in vitro* or/and* in vivo* [11, 12]. Of these, picroliv (*Picrorhiza kurroa*), andrographolide (*Andrographis paniculata*), artemisinin (*Artemisia annua*), and Silymarin extracts for anti-HBV activities are reported long back [13]. Notably, the most promising anti-HBV phytoproducts tested include niranthin and hinokinin (lignans) isolated from* Phyllanthus *spp. [14–16], helioxanthin from the Chinese* Taiwania cryptomerioides* [17], wogonin, another flavonoid from* Scutellaria radix* [18], the polyphenolic extract from* Geranium carolinianum* L. [19], protostane triterpenes from* Alisma orientalis* [20], dihydrochelerythrine alkaloids from* Corydalis saxicola* [21], Saikosaponin C from* Bupleurum* species [22], and extracts from* Rheum palmatum* L. [23]. Very recently, an* in vitro* study showing anti-HBV effect of a compound (LPRP) isolated from* Liriope platyphylla* is also published [24].

*Acacia* is the second largest genus in the Fabaceae family, comprising more than 1200 species worldwide.* A. mellifera* (*AM*), commonly known as “Black Thorn (English)” or “Kekad/Kitir (Arabic)” is widely distributed in Africa and the Arabian Peninsula, including Saudi Arabia [25]. It is a very thorny shrub to small tree with rounded or spreading flat crown which may reach down to ground level. While the twigs are chewed and used as toothbrushes, its pods, young twigs, leaves, and flowers are highly nutritious fodder for livestock.* AM* leaves and roots are used in traditional African ethnomedicine for the treatment of cold, malaria [26], primary infection of syphilis, sterility, and bowel problems [27], including inflammation, diarrhoea, and bleeding [28]. The published reports of various biological activities of* AM* include its antimalarial [29] and antimicrobial [30] potentials. Phytochemical studies on* AM* extracts have indicated that the main components are alkaloids, saponins, flavonoids, tannins, and triterpenoids [31–33]. Of the studied species of* Acacia*,* A. confusa* and* A. auriculiformis* have been reported to have hepatoprotective activity [34, 35]. In addition to this,* A. nilotica* and* A. mellifera* are also shown to have antiviral activities against human immunodeficiency virus-1 (HIV-1) and herpes simplex virus (HSV), respectively [36, 37]. With this background information, we therefore intended to investigate the hepatoprotective as well as anti-HBV activity of organic and aqueous fractions of* AM* leaves extract.

## 2. Materials and Methods

### 2.1. Plant Material

Leaves of* AM* (Family: Fabaceae) were collected from Gezira state, Sudan. Authentication of the plant was confirmed by a taxonomist at the herbarium of the Medicinal and Aromatic Plants Research Institute (MAPRI), Sudan, as well as that of College of Pharmacy, King Saud University, Riyadh, Saudi Arabia. A voucher specimen (number 16281) was deposited at the herbarium of College of Pharmacy, King Saud University.

### 2.2. Extraction and Preparation of* AM* Fractions

The shade-dried and powdered leaves of* AM* (800 g) were soaked in 80% aqueous ethanol (Merck, USA) for two days at 25–30°C and then filtered. Extraction was repeated twice with the same solvent. The extract was collected, passed through Whatmann filter paper number 1 (Whatmann, USA), and then evaporated using a rotary evaporator (Buchi, Switzerland) under reduced pressure at 40°C. The obtained greenish brown semisolid ethanolic-extract (97.47 g) was suspended in distilled water (200 mL) and then fractionated three times successively with the same volume of hexane (Merck, USA), dichloromethane (Merck, USA), ethyl acetate (Merck, USA), and water saturated n-butanol (LobaChemie) to provide the corresponding extracts. The organic solvents of the fractions were evaporated at reduced pressure using rotatory evaporator, and the aqueous extracts were lyophilized in a freeze dryer (Labconco).

### 2.3. Cell Culture and Drug

Human hepatoma cells (HepG2) and HBV cell line, HepG2.2.2.15 (derivative of HepG2) (kind gift of Dr. S. Jameel, International Center for Genetic Engineering & Biotechnology, New Delhi, India), were grown in RPMI-1640 medium (Invitrogen, USA), supplemented with 10% heat-inactivated bovine serum (Gibco, USA), 1x penicillin-streptomycin (Invitrogen, USA), and 1x sodium pyruvate (HyClone Laboratories, USA) at 37°C in a humidified chamber with 5% CO_2_ supply. 2,7-Dichlorofluorescein (DCFH; Sigma, USA) was used as cytotoxin on cultured HepG2 cells. The approved nucleoside analog-based anti-HBV drug, Lamivudine (3TC; Sigma, USA), was used as standard.

### 2.4. Cytotoxicity Assay

*AM* ethanolic-extract as well as the organic fractions was tested for cytotoxic effects, if any, on cultured HepG2 and HepG2.2.15 cells. Cells were seeded (0.5 × 10^5^ cells/well, in triplicate) in a 96-well flat-bottom plate (Becton-Dickinson Labware) and grown over night.* AM* total extract and organic fractions were dissolved in dimethyl sulphoxide (DMSO; 100 mg/mL), followed by dilutions in culture media to prepare five doses (0, 25, 50, 100, and 250 *μ*g/mL) of each. The final concentration of DMSO used never exceeded >0.1% and therefore had no cytotoxicity. The culture monolayers were replenished with complete media containing a dose of* AM* and incubated for 48 h at 37°C followed by MTT assay (TACS MTT Cell Proliferation Assay Kit, Trevigen) as per the manufacturer's instruction. The absorbance/optical density (OD) was recorded at 620 nm in a microplate reader (BioTek, ELx800) and the data analyzed.

### 2.5. Microscopy

A direct visual observation was made under an inverted microscope (Optica, 40x and 100x) to observe any morphological changes in the cells cultured with different concentrations of* AM* fractions and/or DCFH at 24 and 48 h.

### 2.6. * Ex Vivo* Hepatoprotective Activity of* AM* Total Extract and Fractions

HepG2 cells were seeded (0.5 × 10^5^ cells/well, in triplicate) in a 96-well flat-bottom plate (Becton-Dickinson Labware) and grown over night. DCFH prepared (1.0 mg/mL) in DMSO was used as a cytotoxic agent (IC_50_ = 100 *μ*g/mL) (unpublished data).* AM* crude extract and fractions were dissolved in DMSO (100 mg/mL), followed by dilutions in culture media to prepare four doses (25, 50, 100, and 200 *μ*g/mL) of each. The final concentration of DMSO used never exceeded >0.1% and therefore had no cytotoxicity. The culture monolayers were replenished with complete media containing 100 *μ*g/mL of DCFH plus a dose of* AM*, including untreated as well as DCFH-treated controls. The treated cells were incubated for 48 h at 37°C followed by MTT assay. The optical density (OD) was recorded at 620 nm in a microplate reader (BioTek, ELx800) and the data analyzed.

### 2.7. Animals and Treatment

Wister rats (male) were obtained from the Experimental Animal Care Center (EACC) of the College of Pharmacy, King Saud University, Riyadh. Animals were housed in polycarbonate cages in a room free from any source of chemical contamination, artificially illuminated (12 h dark/light cycle) and thermally controlled (25 ± 2°C). After acclimatization, animals were randomized and divided into five groups (I–V) of six animals each. Group I animals served as untreated control and were fed orally with normal saline 1 mL. Group II animals received carbon tetrachloride (CCl_4_) in liquid paraffin (1 : 1; 1.25 mL/kg·bw) intraperitoneally (IP). Groups III, IV, and V received CCl_4_ in liquid paraffin (1 : 1) 1.25 mL/kg·bw. Groups II and III were treated with* AM* total extract at a dose of 250 mg/kg·bw and 500 mg/kg·bw, respectively, for three weeks. Group V was treated with the standard drug Silymarin [38–40] at a dose of 10 mg/kg·bw for three weeks. After collecting the blood, the animals were sacrificed using ether anesthesia. The liver was dissected out and used for biochemical estimations and histological assessment. All animals received human care in compliance with the guidelines of the Ethics Committee of the Experimental Animal Care Society, College of Pharmacy, King Saud University, Riyadh.

### 2.8. Estimation of Marker Enzymes and Bilirubin

Serum glutamate oxaloacetate transaminase (SGOT), serum glutamate pyruvate transaminase (SGPT) [41], alkaline phosphatase (ALP) [42], and gamma-glutamyl transferase (GGT) [43] and bilirubin [44] were determined using Reflotron Plus Analyzer and Roche kits (Roche Diagnostics GmbH, Mannheim, Germany).

### 2.9. Estimation of Lipid Profile

Total cholesterol [45], triglycerides [46], high-density lipoproteins (HDLC) [47], and glucose levels were estimated in serum using Roche diagnostic kits (Roche Diagnostics GmbH, Mannheim, Germany).

### 2.10. Determination of Malondialdehyde (MDA)

The method reported by Utley et al. [48] was followed. In brief, the liver and kidney tissues were removed, and each tissue was homogenized in 0.15 M KCl (at 4°C; Potter-Elvehjem type C homogenizer) to give a 10% (w/v) homogenate. The absorbance of the solution was then read at 532 nm. The content of MDA (nmol/g wet tissue) was then calculated, by reference to a standard curve of MDA solution.

### 2.11. Estimation of Nonprotein Sulfhydryls (NP-SH)

Hepatic NP-SH were measured according to the method described elsewhere [49]. The liver tissues were homogenized in ice-cold 0.02 mM EDTA. The absorbance was measured within 5 min of addition of 5,5′dithio-bis(2)-nitrobenzoic acid (DTNB) at 412 nm.

### 2.12. Determination of Total Protein (TP)

Serum TP was estimated by the kit method (Crescent Diagnostics, Jeddah, Saudi Arabia). The absorbance (Abs) of the complex was measured at 546 nm, and TP was calculated using the standard equation:

(1) Serum  total  protein  =AbssampleAbsstandard×Concentration  of  standard.

### 2.13. Histopathological Evaluation

The animals were sacrificed and dissected liver tissues were fixed in neutral buffered formalin for 24 h. Sections of the liver tissue were histopathologically examined. These sections were stained with haematoxylin and eosin using routine procedures [50].

### 2.14. Phytochemical Screening for Secondary Metabolites

Qualitative phytochemical screening of* AM* total extract and its fractions for major secondary metabolites, like alkaloids, flavonoids, anthraquinones, tannins, and saponins, was performed using standard procedures as described elsewhere [51–53].

### 2.15. *In Vitro* Antioxidant Activities of* AM* Fractions

The antioxidant activity of the fractions was evaluated using the *β*-carotene-linoleic acid bleaching method with minor modifications for working with 96-well plate. Briefly, 0.5 mg *β*-carotene (Sigma Aldrich, USA) was dissolved in 1 mL of chloroform (Merck, USA) and added to flasks containing 25 *μ*g of linoleic acid (Sigma Aldrich, USA) and 200 mg of Tween-40 (Sigma Aldrich, USA). The chloroform was removed at 40°C using a rotary evaporator. The resultant mixture was immediately diluted with 50 mL of distilled water and mixed for 1-2 min to form an emulsion. A mixture prepared similarly but without *β*-carotene was used as a blank, including a second control containing solvent instead of extract. A 200 *μ*L aliquot of the emulsion was added to wells of 96-well plate containing 40 *μ*L of the test sample (in triplicate) and 500 *μ*g/mL of gallic acid (Sigma Aldrich, USA) was used as a standard. The plate was incubated at 50°C for 2 h and absorbance was read (490 nm) at 30 min intervals using microplate reader (BioTek, ELx800). The antioxidant activity was calculated using the following equation:

(2) $$\begin{array}{c} \%\text{Antioxidant}\;\text{activity}=1-\frac{\left({\text{Abs}}_{0}-{\text{Abs}}_{t}\right)}{\left({\text{Abs}}_{0}^{o}-{\text{Abs}}_{t}^{o}\right)}\times 100, \end{array}$$

where Abs_0_ and Abs_0_^*o*^ are the absorbance values measured at zero time of incubation for sample extract and control, respectively. Abs_*t*_ and Abs_*t*_^*o*^ are the absorbance values for sample extract and control, respectively, at *t* = 120 min.

### 2.16. *In Vitro* Free Radical Scavenging Activity of* AM* Fractions

The free radical scavenging ability of the different* AM* fractions against 1,1-diphenyl-2-picrylhydrazyl (DPPH) radical (Sigma Aldrich, USA) was evaluated as previously described method [54] with minor modifications.* AM* total extract and fractions were dissolved in DMSO (100 mg/mL), followed by dilutions with methanol to various concentrations (25, 50, 100, and 500 *μ*g/mL). Each fraction (150 *μ*L) was mixed with 50 *μ*L of DPPH (0.004% w/v in methanol) in triplicate in a 96-well plate. Appropriate blanks were prepared using the solvent only in addition to the same amount of DPPH reagent to get rid of any inherent solvent effect. Ascorbic acid was used as standard. After 30 min of incubation at 25°C, the decrease in absorbance was measured (490 nm). The radical scavenging activity was calculated from the following equation:

(3) %Radical  scavenging  activity  =Abscontrol−AbssampleAbscontrol×100.

### 2.17. Dose-Dependent Analysis of Anti-HBV Activities of* AM* Fractions

The HBV cell line, HepG2.2.15, was seeded in 96-well plates (0.5 × 10^5^/well in triplicate), including naïve HepG2 cells as negative control. Next day, the old media were replaced with 100 *μ*L each of four doses (31.25, 62.5, 125 and 250 *μ*g/mL prepared in culture media) of the five* AM* fractions, including Lamivudine (2.0 *μ*M), and the culture was incubated for 2 days. Culture supernatants of each sample (triplicates) were collected and analyzed for the viral HBsAg and HBeAg using Monolisa HBsAg ULTRA Elisa Kit (BioRad, USA) and HBeAg/Anti-HBe Elisa Kit (DIASource, Belgium), respectively, as per the manufacturer's manual.

### 2.18. Time-Course Analysis of HBsAg and HBeAg Expressions

Further antiviral activities of the most active fractions at the highest dose were tested at days 1, 3, and 5. Analysis of inhibition of HBsAg and HBeAg secretions in the culture supernatants was done as mentioned above.

## 3. Results

### 3.1. Effect of* AM* Crude Ethanolic-Extract on Cell Morphology and Growth

DCFH showed considerable cytotoxic effect on the HepG2 and HepG2.2.15 cells as reflected by altered morphology compared to untreated cells. Interestingly, the DCFH-treated cells supplemented with 100 *μ*g/mL and 200 *μ*g/mL of* AM* crude ethanolic extract and fractions were morphologically different from the DCFH-treated cells but comparable to untreated cells at 48 h (data not shown).

### 3.2. Hepatoprotective Effect of* AM* Organic Fractions on Cultured Liver Cells

**Figure 1:**
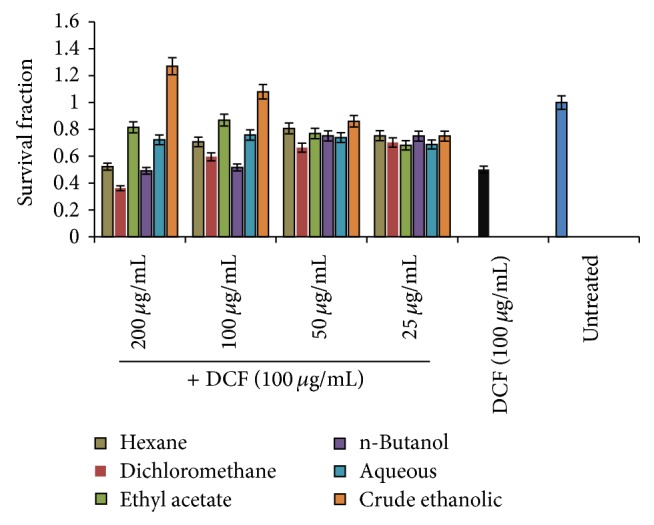
MTT Cell Proliferation Assay. Hepatoprotective effect of* A. mellifera* (*AM*) crude ethanolic extract and five fractions (hexane, dichloromethane, ethyl acetate, n-butanol, and aqueous) against DCFH-induced toxicity of HepG2 cells.

Hepatoprotective effect of* AM* crude extract and fractions against DCFH-induced hepatotoxicity was investigated. While DCFH-toxicated cells were recovered to about 100% with 100 *μ*g/mL of* AM* crude extract, supplementation with 200 *μ*g/mL of this further enhanced the hepatocytes proliferation by about 20% (Figure 1). Of the five fractions evaluated, the ethyl acetate, aqueous and n-butanol fractions showed the most effective hepatoprotection (Figure 1).

### 3.3. *In Vivo* Effect of* AM* Crude Extract on Biochemical Markers

Based on the hepatoprotective activity at cellular level, the effect of* AM* extract was further examined in the animal model. Administration of CCl_4_ dramatically elevated the sera AST, ALT, GGT, and ALP and bilirubin levels compared to the normal control group (*P* < 0.0001), indicating significant hepatotoxicity of CCl_4_ treatment (Table 1). In contrast, administration of* AM* extract significantly decreased the above elevated parameters in CCl_4_-treated rats compared to the CCl_4_-treated group. Moreover, CCl_4_-induced toxicity caused significant elevation in lipid profile including cholesterol, triglycerides, LDL-C, and VLDL-C and reduction in the HDL-C levels in serum. After three weeks, while* AM* extract in a dose-dependent manner significantly reduced the cholesterol, triglycerides, LDL-C, and VLDL-C levels, it greatly improved HDL-C level (Table 2). Silymarin used as standard, on the other hand, significantly normalized the CCl_4_-induced elevated levels of marker enzymes and lipids. Furthermore, our results indicated that treatment with CCl_4_ resulted in a significant increase in MDA but decrease in NP-SH and TP concentration (Table 3). Treatment of rats with* AM* extract resulted in a significantly diminished level of MDA and greatly enhanced NP-SH and TP levels.

### 3.4. Histological Improvement of Injured Liver by* AM*

**Figure 2:**
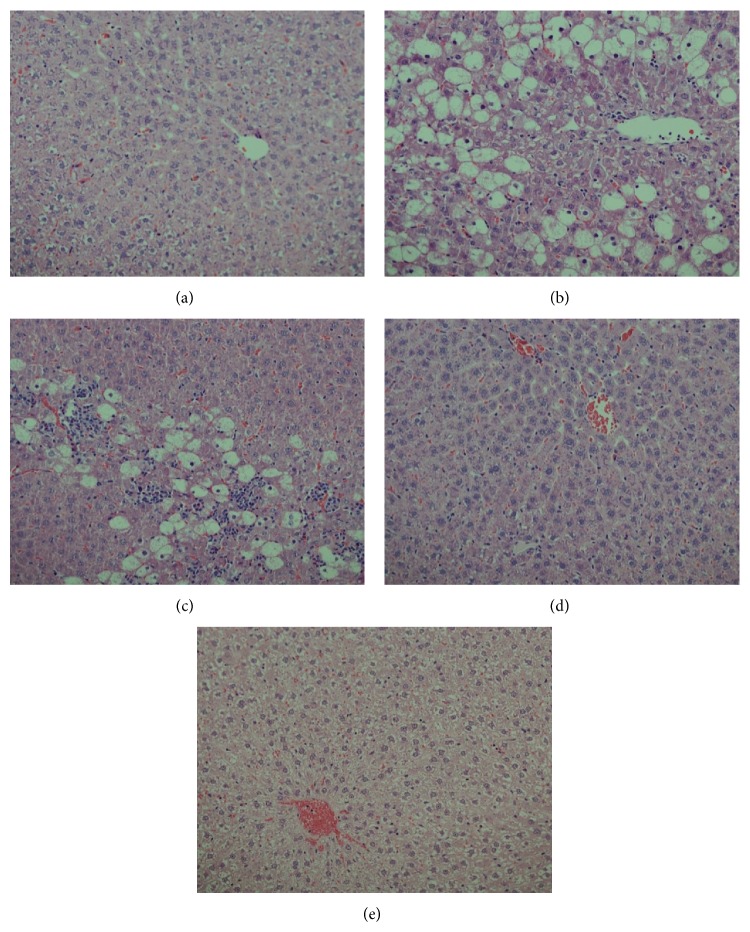
Histopathology of experimental rat liver. Histograms showing (a) healthy tissues with normal hepatocytes and central vein, (b) CCl_4_-injured tissue with necrosis and fatty degenerative changes, (c) tissue with congested central vein with necrosis and fatty changes after 250 mg of* A. mellifera* (*AM*) + CCl_4_ treatment, (d) liver with normal hepatocytes and central vein with full recovery after 500 mg of* AM* + CCl_4_ treatment, and (e) liver with normal hepatocytes and fully recovered central vein after Silymarin + CCl_4_ treatment.

The histological examination of rat liver tissues revealed evidence of hepatic necrosis and fatty degenerative changes in CCl_4_-injured animals. Compared to this, the* AM* extract-treated (250 mg/kg/day) animals exhibited congested central vein with mild necrosis and fatty changes. On the other hand, the higher dose (500 mg/kg/day) of* AM* or Silymarin administration showed normal hepatocytes and central vein with full recovery (Figures 2(a)–2(e)). This finally confirmed the* in vivo *hepatoprotective efficacy of* AM* that supported our* ex vivo* data.

### 3.5. Phytochemical Screening

The qualitative phytochemical screening of the* AM* crude extract and organic fractions showed the presence of alkaloids, flavonoids, polyphenolic tannins, sterols, and saponins. There was, however, no evidence of anthraquinones in the fractions.

### 3.6. Antioxidant Activity of* AM* Fractions

The crude ethanolic extract of* AM* was able to reduce the stable free radical DPPH to the yellow-colored DPPH at low concentrations (100 and 500 *μ*g/mL), almost near to the positive control. Moreover, in BCBT, the extract was also able to inhibit the discoloration of *β*-carotene at a concentration of 500 *μ*g/mL. The total estimated antioxidant value was 87% comparable to that of positive control. Based on this result, the four organic fractions were further tested for antioxidant activity (Table 4). The highest antioxidant activity was found in the ethyl acetate and n-butanol extracts following dichloromethane and hexane extracts. The aqueous extract was found to have the least activities.

### 3.7. Inhibition of HBsAg Expression by* AM* n-Butanol and Aqueous Fractions

**Figure 3:**
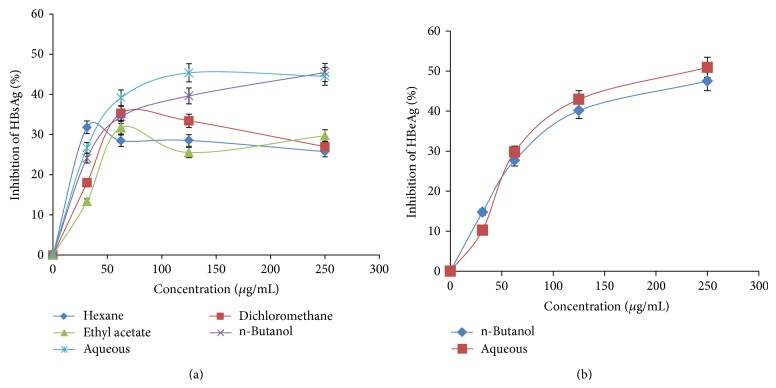
Dose-dependent anti-HBV activities of* A. mellifera* (*AM*) leaves extracts. ELISA showing inhibitions of (a) HBsAg expression by* AM* organic and aqueous fractions and (b) HBeAg expression by* AM* n-butanol and aqueous fractions in HepG2.2.15 culture supernatants. Doses used for* AM*: 31.25, 62.5, 125, and 250 *μ*g/mL. Values (*y*-axis): means of 3 determinations.

**Figure 4:**
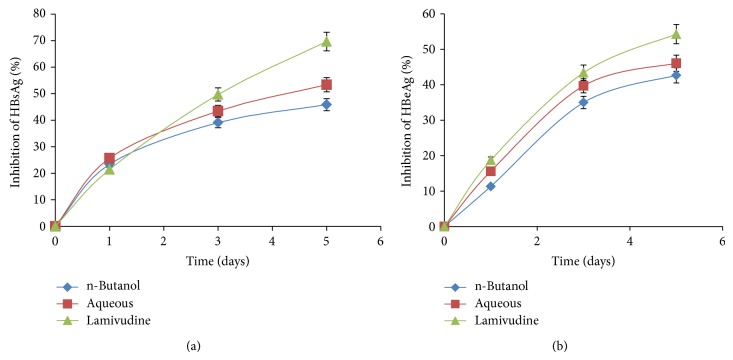
Time-course anti-HBV activities of* A. mellifera* (*AM*) n-butanol and aqueous fractions (125 *μ*g/mL each). ELISA showing inhibitions of (a) HBsAg expression and (b) HBeAg expression in HepG2.2.15 culture supernatants at days 1, 3, and 5. Lamivudine (2.0 *μ*M) used as reference anti-HBV drug. Values (*y*-axis): means of 3 determinations.

Dose- and time-dependent activities of five fractions of* AM* extract were tested for inhibition of expression levels of viral HBsAg with reference to untreated controls. At day 2 after treatment, while the hexane, dichloromethane, and ethyl acetate fractions showed about 25–35% of downregulation of HBsAg expressions at highest doses (125 and 250 *μ*g/mL), the n-butanol and aqueous fractions exhibited the best inhibitions by approximately ~46% and ~44%, respectively (Figure 3(a)). Inhibitory effects of n-butanol and aqueous fractions were further evaluated in a time-course study, using 125 *μ*g/mL dose. Compared to days 1 and 3 post-treatment, HBsAg production was inhibited up to ~50% and ~40% by n-butanol and aqueous fraction, respectively on day 5 (Figure 4(a)). While prolonged treatment beyond day 5 did not show any significant difference, further continuation of culture resulted in cell overgrowth and death (data not shown).

### 3.8. Downregulation of HBV Replication by* AM* n-Butanol and Aqueous Fractions

The HBV “e” antigen (secretory protein) is a processed product of “pre-Core” that is cotranslated with “Core” by a bicistronic subgenomic-RNA. Therefore, production of HBeAg is a hallmark of HBV DNA replication (except HBeAg negative chronic hepatitis B cases). This is analogous to HIV-p24 antigen where ELISA is a valid tool to monitor HIV replication. Therefore, the two most promising* AM* fractions, n-butanol and aqueous, that greatly suppressed HBsAg synthesis were subjected to HBeAg analysis of the culture supernatants. The two fractions very clearly showed inhibition of HBeAg production in a dose-dependent manner. At day 2 after treatment, while 125 and 250 *μ*g/mL of n-butanol fraction downregulated HBV replication by ~40% and ~48%, respectively, those of aqueous fraction inhibited virus replication by ~41% and ~50%, respectively (Figure 3(b)). Replication inhibitory effects of n-butanol and aqueous fractions were further evaluated in a time-course study, using the 125 *μ*g/mL dose. Compared to days 1 and 3 post-treatment, HBeAg production was inhibited up to ~50% and ~40% by n-butanol and aqueous fractions, respectively on day 5 (Figure 4(b)). While prolonged treatment beyond day 5 did not show any significant difference, further continuation of culture resulted in cell overgrowth and death (data not shown).

## 4. Discussion

In the present study, we first evaluated the hepatoprotective effects of* AM* total ethanolic-extract and its five fractions against the DCFH-induced cytotoxicity on HepG2 cells. DCFH is generally used to measure* in vitro* oxidative stress generated by free radicals through the principle of oxidation of DCFH to the fluorescent DCF [55]. However, we used this agent because of its cytotoxic effect* in vitro*.* AM* organic fractions not only protected the cells against DCFH-induced toxicity, but also promoted cell recovery and proliferation. These findings were in line with our visual examination of the cell morphology under microscope. The total ethanol extract was, however, more active than the hexane, dichloromethane, ethyl acetate, n-butanol, and aqueous fractions indicating synergy between different secondary metabolites.

CCl_4_ is a well-known hepatotoxin and is frequently used as a chemical inducer of liver damage. Its metabolic transformation by cytochrome P^450^ to trichloromethyl and trichloromethyl peroxyl free radicals causes covalent binding to macromolecules and lipid peroxidation, resulting in cell injury [2, 3]. Liver damage by acute exposure to CCl_4_, therefore, causes clinical symptoms such as jaundice and elevated levels of liver enzymes in the blood [53]. The liver enzymes such as AST, ALT, and ALP found within organs and tissues are released into the bloodstream following cellular necrosis and cell membrane permeability and are used as a diagnostic indicator of liver damage* in vivo*. In the present study, treatment of CCl_4_-injured rats with 250 and 500 mg/kg of* AM* total extract significantly reduced the sera ALT, AST, GGT, and ALP and bilirubin levels in a dose-dependent manner. Similar trend was observed for the sera cholesterol, TG, and HLD levels. The effect of* AM* extract was comparable to Silymarin used as standard drugs that suggested a hepatoprotective effect of* AM* extract* in vivo*. Further, the significant reduction in levels of LDLP-C, LDLP-C, and total cholesterol in the* AM*-treated rats and an increase in HDL-C level supported the hepatoprotective potential of the* AM* extract.

MDA is a metabolite that is produced during lipid peroxidation of cell membrane and is used as an indicator for cell damage [56]. The level of MDA was reduced in both* AM*- and Silymarin-treated rats which also suggested the protective and curative activities of* AM* against liver damages. NPSH are involved in several defense processes against oxidative damage. In the current study, the liver NP-SH levels in CCl_4_-treated rats were significantly diminished when compared with the control group. Treatment of CCl_4_-injured rats with* AM* or Silymarin replenished NPSH concentration compared to untreated animals that further suggested free radical scavenging activity of* AM* extract* in vivo*. Moreover, the levels of TP in rat serum were related to the function of hepatocytes. Diminution of TP is a further indication of liver damage in CCl_4_-injured animals. The level of TP would be decreased in hepatotoxic condition due to defective protein biosynthesis in liver. In our study, the level of TP was restored to normal value indicating its hepatoprotective activity of* AM* that was comparable to Silymarin.

*In vitro *antioxidant activity of* AM* total ethanolic-extract and organic fractions revealed strong antioxidant activity in DPPH test. The antioxidant activity could be thus attributed to the presence of antioxidant and free radical scavenging factors, for example, phenolic compounds, flavonoids, and saponins, which were reported to have hepatoprotective activity [57–60]. Hepatoprotective activity of flavonoids is due to their ability to scavenge free radicals [58]. Such high activity observed for ethyl acetate and n-butanol fractions compared to the corresponding hexane, dichloromethane, and aqueous fractions suggested that the antioxidant compound(s) is of high to intermediate polarity. Furthermore, the higher activity observed for n-butanol fraction compared to the aqueous one can be explained. Most of the active phenolic compounds and glycosides were taken from aqueous phase by n-butanol and metal salts, which, if present, could be dissolved in the aqueous phase that could have inhibited the activity of antioxidants [61]. Nevertheless, there is a linear relationship between the hepatoprotective and the antioxidant activity. HBV and HIV share some biological properties, including mechanism of genome replication. While HIV and HSV are retroviruses, HBV is called pararetrovirus that replicates its DNA genome through a unique RNA intermediate step using reverse-transcriptase. Therefore, many of the licensed drugs originally developed for HIV and HSV have been also effective against HBV. The antiviral activities of* A. mellifera* and the aqueous extract of* A. nilotica* against HSV and HIV, respectively, are previously reported [36, 37]. In line with this, our anti-HBV evaluations of the different fractions at noncytotoxic concentrations, the n-butanol and aqueous fractions, showed very promising antiviral activities. The variable activities shown by the different fractions may be attributed to the diversity of structure and/or uneven distribution of phytochemical constituents present in these fractions. Nevertheless, this further suggests that the bioactive compounds present in tested* AM* fractions are of high polarity. Identification of alkaloids, flavonoids, and polyphenols showing antioxidant and anti-HBV activities in our study is in accordance with previously published reports.

## 5. Conclusions

Our results revealed very promising hepatoprotective and cell-proliferative effects of* AM* leaves fractions in both* ex vivo* and* in vivo* experimental conditions. Interestingly, further* ex vivo* evaluations of the n-butanol and aqueous fractions also exhibited anti-HBV efficacy. Taken together, while the ethyl acetate and aqueous fractions exhibited the most promising antioxidant/hepatoprotective and anti-HBV activity, respectively, the n-butanol fraction showed both activities. Therefore, the therapeutic potential of* AM* extracts warrants further isolation of the active principle(s) and its phytochemical as well as biological studies.

## Figures and Tables

**Table 1:** *In vivo* effect of *A. mellifera *(*AM*) crude ethanolic extract on CCl_4_-induced hepatotoxicity-related parameters.

Treatment group	Dose mg/kg	AST (U/L)	ALT (U/L)	GGT (U/L)	ALP (U/L)	Bilirubin (mg/dL)
Normal		106.15 ± 4.36	37.91 ± 1.61	3.26 ± 0.21	308.83 ± 8.81	0.5 ± 0.02
CCl_4_	1.25	379.83 ± 11.70^∗∗∗a^	303.83 ± 12.12^∗∗∗a^	18.20 ± 0.89^∗∗∗a^	586.16 ± 11.92^∗∗∗a^	2.74 ± 0.1^∗∗∗a^
AM + CCl_4_	250	322.50 ± 12.08^∗∗b^	264.83 ± 10.74^∗b^	12.41 ± 0.73^∗∗∗b^	512.0 ± 11.40^∗∗b^	1.66 ± 0.10^∗∗∗b^
AM + CCl_4_	500	283.66 ± 9.82^∗∗∗b^	178.16 ± 6.27^***^	6.90 ± 0.38^∗∗∗b^	398.50 ± 0.48^∗∗∗b^	1.16 ± 0.10^∗∗∗b^
Silymarin + CCl_4_	10	166.16 ± 9.34^b^	99.75 ± 3.55^***^	5.78 ± 0.26^∗∗∗b^	325 ± 33 ± 12.10^∗∗b^	0.94 ± 0.04^∗∗b^

All values represent mean ± SEM. ^*^ *P* < 0.05; ^**^ *P* < 0.01; ^***^ *P* < 0.001; ANOVA, followed by Dunnett's multiple comparison test. ^a^As compared with normal group. ^b^As compared with CCl_4_ only group.

**Table 2:** *In vivo* effect of *A. mellifera *(*AM*) crude ethanolic extract on CCl_4_-induced lipid profile changes.

Treatment group	Dose mg/kg	TC (mg/dL)	TG (mg/dL)	HDL-C (mg/dL)	LDL-C (mg/dL)	VLDL-C (mg/dL)
Normal		88.09 ± 6.5	78.26 ± 4.09	50.82 ± 1.25	72.24 ± 6.34	15.56 ± 0.65
CCl_4_	1.25 mL/kg	212.69 ± 7.84^∗∗∗a^	184.05 ± 8.09^∗∗∗a^	22.14 ± 0.56^∗∗∗a^	175.80 ± 8.42^∗∗∗a^	36.81 ± 1.69^∗∗∗a^
AM + CCl_4_	250	169.04 ± 11.25^∗∗∗b^	168.59 ± 9.17^b^	28.26 ± 1.25^∗∗b^	135.22 ± 10.18^∗b^	33.71 ± 1.83^b^
AM + CCl_4_	500	136.50 ± 4.70^∗∗∗b^	117.37 ± 7.01^∗∗∗b^	36.76 ± 1.02^∗∗∗b^	112.89 ± 4.02^∗∗∗b^	23.47 ± 1.40^∗∗∗b^
Silymarin + CCl_4_	10	124.60 ± 5.94^∗∗∗b^	98.55 ± 5.60^∗∗∗b^	35.34 ± 1.81^∗∗∗b^	104.74 ± 5.93^∗∗∗b^	19.71 ± 1.12^∗∗∗b^

All values represent mean ± SEM. ^*^ *P* < 0.05; ^**^ *P* < 0.01; ^***^ *P* < 0.001; ANOVA, followed by Dunnett's multiple comparison test. ^a^As compared with normal group. ^b^As compared with CCl_4_ only group.

**Table 3:** Biochemical parameters of rat liver after treatment with *A. mellifera *(*AM*) crude ethanolic extract.

Treatment group	Dose mg/kg	TP (mg/dL)	MDA (nmol/g)	NP-SH (mg/dL)
Normal		95.84 ± 6.27	0.96 ± 0.11	8.16 ± 0.42
CCl_4_	1.25 mL/kg	30.78 ± 3.13^∗∗∗a^	8.55 ± 1.07^∗∗∗a^	4.25 ± 0.42^∗∗∗a^
AM + CCl_4_	250	36.21 ± 3.22^b^	5.57 ± 0.77^∗b^	6.20 ± 0.56^∗b^
AM + CCl_4_	500	56.63 ± 3.80^∗∗∗b^	2.257 ± 0.20^∗∗∗b^	6.28 ± 0.48^∗b^
Silymarin + CCl_4_	10	56.64 ± 7.61^∗∗∗b^	2.87 ± 0.64^∗∗∗b^	7.36 ± 0.54^∗∗b^

All values represent mean ± SEM. ^*^ *P* < 0.05; ^**^ *P* < 0.01; ^***^ *P* < 0.001; ANOVA, followed by Dunnett's multiple comparison test. ^a^As compared with normal group. ^b^As compared with CCl_4_ only group.

**Table 4:** Free radical scavenging and total antioxidant activities of *A. mellifera *(*AM*) five fractions.

*AM* fractions	Radical scavenging activity (%) (DPPH)				Total antioxidant activity (%) (BCBT)
	25 (*µ*g/mL)	50 (*µ*g/mL)	100 (*µ*g/mL)	500 (*µ*g/mL)	500 (*µ*g/mL)
Hexane	—^**^	—	4.017 ± 0.07^*^	6.83 ± 0.05	55.51 ± 0.98
Dichloromethane	8.97 ± 1.24	10.15 ± 1.15	27.90 ± 1.87	44.39 ± 0.04	69.75 ± 1.12
Ethyl acetate	30.00 ± 0.04	44.167 ± 1.03	65.83 ± 1.08	86.66 ± 1.81	78.16 ± 1.03
n-Butanol	20.51 ± 1.34	38.20 ± 0.07	62.53 ± 0.42	77.50 ± 0.14	72.33 ± 0.81
Aqueous	—	0.67 ± 0.05	7.25 ± 0.02	38.87 ± 1.06	62.30 ± 0.57
Ascorbic acid	90.97 ± 0.07	92.13 ± 0.28	92.15 ± 0.06	92.40 ± 0.32	—
Gallic acid	—	—	—	—	87.12 ± 1.05

^*^ Mean ± standard error of mean (SEM) for triplicate experiments.

^**^Not available.
